# Acute Adverse Effects of Fine Particulate Air Pollution on Ventricular Repolarization

**DOI:** 10.1289/ehp.0901648

**Published:** 2010-04-02

**Authors:** Duanping Liao, Michele L. Shaffer, Sol Rodriguez-Colon, Fan He, Xian Li, Deborah L. Wolbrette, Jeff Yanosky, Wayne E. Cascio

**Affiliations:** 1 Department of Public Health Sciences and; 2 Department of Medicine, Division of Cardiology, Penn State University College of Medicine, Hershey, Pennsylvania, USA; 3 Department of Cardiovascular Sciences, Brody School of Medicine, and the East Carolina Heart Institute at East Carolina University, Greenville, North Carolina, USA

**Keywords:** autonomic modulation, cardiovascular disease, particulate matter, QT interval, ventricular repolarization

## Abstract

**Background:**

The mechanisms for the relationship between particulate pollution and cardiac disease are not fully understood.

**Objective:**

We examined the effects and time course of exposure to fine particulate matter ≤ 2.5 μm in aerodynamic diameter (PM_2.5_) on ventricular repolarization of 106 nonsmoking adults who were living in communities in central Pennsylvania.

**Methods:**

The 24-hr beat-to-beat electrocardiogram (ECG) data were obtained using a high-resolution 12-lead Holter system. After visually identifying and removing artifacts and arrhythmic beats, we summarized normal beat-to-beat QTs from each 30-min segment as heart rate (HR)-corrected QT measures: QT prolongation index (QTI), Bazett’s HR-corrected QT (QTcB), and Fridericia’s HR-corrected QT (QTcF). A personal PM_2.5_ monitor was used to measure individual-level real-time PM_2.5_ exposures for 24 hr. We averaged these data and used 30-min time-specific average PM_2.5_ exposures.

**Results:**

The mean age of the participants was 56 ± 8 years, with 41% male and 74% white. The means ± SDs for QTI, QTcB, and QTcF were 111 ± 6.6, 438 ± 23 msec, and 422 ± 22 msec, respectively; and for PM_2.5_, the mean ± SD was 14 ± 22 μg/m^3^. We used distributed lag models under a framework of linear mixed-effects models to assess the autocorrelation-corrected regression coefficients (β) between 30-min PM_2.5_ and the HR-corrected QT measures. Most of the adverse ventricular repolarization effects from PM_2.5_ exposure occurred within 3–4 hr. The multivariable adjusted β (SE, *p*-value) due to a 10-μg/m^3^ increase in lag 7 PM_2.5_ on QTI, QTcB, and QTcF were 0.08 (0.04, *p* < 0.05), 0.22 (0.08, *p* < 0.01), and 0.09 (0.05, *p* < 0.05), respectively.

**Conclusions:**

Our results suggest a significant adverse effect of PM_2.5_ on ventricular repolarization. The time course of the effect is within 3–4 hr of elevated PM_2.5_.

Numerous studies have consistently found a significant association between fine particulate matter ≤ 2.5 μm in aerodynamic diameter (PM_2.5_) air pollution and the short-term risk of clinical cardiovascular mortality (Franklin et al. 2007, 2008; Ostro et al. 2006; Zanobetti and Schwartz 2009). The mechanisms responsible for such an association have been the focus of recent environmental health studies. In population-based studies of healthy individuals (Dekker et al. 1994; Goldberg et al. 1991; Peters et al. 1990; Rautaharju et al. 2006a, 2006b; Schouten et al. 1991), longer repolarization within normal range was significantly associated with cardiac events, especially sudden cardiac death. Similar findings from clinical populations (Siscovick et al. 1996; Whitsel et al. 2000, 2001) have also been reported. Recent studies have suggested that one of the underlying mechanisms linking air pollution and increased risk of cardiovascular disease (CVD) is the effect of PM on ventricular repolarization (Campen et al. 2006; Ghelfi et al. 2008; Henneberger et al. 2005; Lux and Pope 2009; Samet et al. 2009; Yue et al. 2007). For the time course of PM effects on cardiac electrophysiology, several published studies have suggested shorter time effects, such as within the same day or 1–2 days prior to electrocardiogram (ECG) measurements (Elder et al. 2007; Liao et al. 1999, 2004, 2009; Lux and Pope 2009; Park et al. 2005; Yue et al. 2007; Zanobetti et al. 2009; Zhang et al. 2009). For patients who wore implanted cardioverter defibrillators, Dockery et al. (2005) reported significantly increased incidence of arrhythmias associated with the 2-day average of various pollutants, which also suggested acute effects of pollution on clinically relevant arrhythmias. In one study specifically designed to investigate the time course of PM on heart rate variability (HRV), Cavallari et al. (2008) reported an early- and a later-phase response, with the early effects at 2 hr and delayed effects at 9–13 hr after exposure.

We therefore designed this study to investigate the effects and time course of individual-level exposures to PM_2.5_ on the ventricular repolarization in a sample of nonsmoking adults who lived in communities in central Pennsylvania.

## Materials and Methods

### Population

For this report, we used the data collected for the Air Pollution and Cardiac Risk and its Time Course (APACR) study, which we designed to investigate the mechanisms and the time course of the adverse effects of PM_2.5_ on cardiac electrophysiology, blood coagulation, and systemic inflammation. The APACR study has maintained approval by Penn State University College of Medicine institutional review board. All participants gave written informed consent prior to their participation in the study. All study participants were recruited from communities in central Pennsylvania, mostly from the Harrisburg metropolitan area. The inclusion criteria for the study included nonsmoking adults > 45 years old who had not been diagnosed with severe cardiac problems (defined as diagnosed valvular heart disease, congenital heart disease, acute myocardial infarction or stroke within 6 months, or congestive heart failure). Community recruitment specialists from the General Clinical Research Center (GCRC), which is funded by the National Institutes of Health, at the Penn State College of Medicine, and the GCRC-organized community outreach activities, supported the recruitment of the participants. The GCRC maintains a list of individuals who live in central Pennsylvania communities for various health-related studies. The APACR study participants were numerated from the GCRC’s list of potential participants; approximately 75% of the individuals who were contacted and who met our inclusion criteria were enrolled in the study. Our targeted sample size was 100 individuals, and we enrolled and examined 106 individuals. The examination of two participants per week was conducted from November 2007 to June 2009 for the entire examination period except for major holidays.

Study participants were examined in the GCRC in the morning between 0800 and 1000 hours. All participants fasted for at least 8 hr before the clinical examination. After completing a health history questionnaire, a trained research nurse measured seated blood pressure (BP) three times, height, and weight, and drew 50 mL blood for biomarker assays according to the blood sample preparation protocols. A trained investigator connected the PM_2.5_ and Holter ECG recorders. Participants were given an hourly activity log to record special events that occurred in the next 24 hr, including outdoor activities, exposure to traffic on the street, travel in an automobile, and any physical activities. The entire examination session lasted for about 1 hr. Participants were then released to proceed with their usual daily routines. The next morning, they returned to the GCRC to remove the PM and Holter monitors, to deliver the completed activity log, and to have their seated BP measured three times and another 50 mL of blood drawn. An exercise echocardiogram was then performed to measure the ventricular function and structure for each participant. The entire second day session lasted for about 1 hr and 45 min. A description of the participants’ characteristics are presented in Table 1.

The study protocol was approved by Penn State University College of Medicine institutional review board. Each participant received $50 and two certificates for breakfast in the hospital cafeteria, and they were reimbursed for their transportation costs.

### PM_2.5_ concentration

The APACR study used personal PM_2.5_ DataRam (pDR; Thromo Scientific, Boston, MA) for real-time 24-hr personal PM_2.5_ exposure assessment. The pDR used light-scattering physics of the fine particles to detect the real-time concentrations of particles of various sizes. The size selection was achieved by using an active pump with a validated size-selection cyclone inlet (KTL SCC1.062; BGI Inc., Waltham, MA) at a flow rate of 1.5 L/min. The standardized operation procedures (SOP) for the use of the pDR, including the calibration, application, data transfer, and data validation, as well as chemical analysis of filters for major PM_2.5_ species, were developed by the APACR investigators (Penn State University 2008a). The standardized procedures were rigorously followed in the PM_2.5_ data collection. The real-time PM_2.5_ concentrations were initially recorded at 1-min intervals. For each participant, we calculated the 30-min segment-specific averages, based on the top and the bottom of the clock time, as our PM_2.5_ exposure variable in the APACR study. Therefore, the PM_2.5_ exposure variables were treated as repeated measures, and each individual contributed 48 exposure data points.

### Continuous ambulatory ECG

A high-fidelity (sampling frequency 1,000 Hz) 12-lead HScribe Holter System (Mortara Instrument, Inc., Milwaukee, WI) was used to collect the 24-hr Holter beat-to-beat ECG data. The high-fidelity ECG significantly increases the resolution and enhances the accuracy of various wave form measurements. The Holter ECG data were scanned to a designated computer for offline processing by an experienced investigator using specialized SuperECG software (Mortara Instrument, Inc.). The SOPs for the APACR study were developed by the study investigators (Penn State University 2008b, 2008c) and were rigorously followed in the data collection and interpretation processes. Briefly, the Holter ECG Data Collection and Analysis Procedures (Penn State University 2008b) were followed to prepare, hook up, calibrate, and start the Holter digital recorder. After 24 hr of recording, a trained investigator followed the SOP to retrieve and archive the beat-to-beat ECG data for offline processing. The main objective of the offline processing was to verify the Holter-identified ECG waves and to identify and label additional electronic artifacts and arrhythmic beats in the ECG recording. Finally, a single research investigator followed the SuperECG Manual (Penn State University 2008c) to perform beat-to-beat ECG analysis to calculate ECG parameters.

### QT interval and QRS duration variables

We used the above described 24-hr beat-to-beat Holter ECG, after removing artifacts with standardized visional inspection and statistical filters, to calculate beat-to-beat QT intervals using the SuperECG software, which defined QT interval as the start of Q wave to the end of T wave. None of the normal sinus QRS durations was > 120 msec. QT is heart rate (HR) dependent, and only after HR correction does the QT interval have the electrophysiological property of ventricular repolarization. We then calculated the following three HR-corrected QT duration indices as the measures of ventricular repolarization on a 30-min basis.

- Prolongation index (QTI) = 100 × (QT_max_ ÷ QT_predicted_), where QT_max_ is the maximum QT duration across all normal cardiac cycles within the segment and QT_predicted_ = 656 ÷ (1 + 0.01 × HR) (Rautaharju et al. 1991).
- Bazett’s HR-corrected QT interval (QTcB) (Bazett 1920) = 
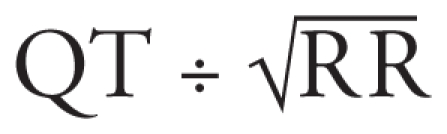
.
- Fridericia’s HR-corrected QT interval (QTcF) (Fridericia 2002) = 
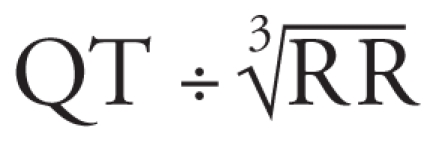
.

These HR-corrected indices were chosen because the QTI has been reported to be (Rautaharju et al. 1991) less rate sensitive and because it has a higher rate of repeatability than does QT_c_B, which is the first HR-correction QT index; QTcF has similar properties but better HR correction compared with QTcB. These ECG measures were treated as repeated outcome measures, and each individual contributed 48 outcome data points on each of the three ventricular repolarization variables.

The QRS duration (QRS) was calculated as the duration from the start of Q wave to the end of S wave. QRS duration was used as a measure of ventricular depolariation.

### HRV variables

We performed time and frequency domain HRV analysis on the ECG recording after removing artifacts with standardized visional inspection and statistical filters. We calculated HRV indices from overall 24-hr recording, 30-min, and 5-min segment-specific recordings using the SuperECG package (Mortara Instrument, Inc.) according to the current recommendations (Task Force of the European Society of Cardiology and the North American Society of Pacing and Electrophysiology 1996). The following HRV indices were used as indices of cardiac autonomic modulation: SD of all RR intervals (SDNN, milliseconds), square root of the mean of the sum of the squares of differences between adjacent RR (RMSSD, msec), power in the low-frequency (LF) range (0.04–0.15 Hz), power in the high frequency (HF) range (0.15–0.40 Hz), and the ratio of LF and HF.

### Weather variables

We obtained real-time temperature and relative humidity using the HOBO H8 logger (Onset Computer Corporation, Bourne, MA). The real-time temperature and relative humidity were recorded at 1-min intervals initially. For each participant, we calculated 30-min segment-specific averages, corresponding to the PM_2.5_ and Holter measures. Therefore, these weather covariables were treated as repeated measures, and each individual contributed 48 data points for each variable.

### Other participant-level covariables

A standardized questionnaire administered on day 1 of the study was used to collect the following individual-level information: *a*) demographic variables, including age, race, sex, and highest education level; *b*) medication uses, including antianginal medication, antihypertensive medication, and antidiabetic medication; and *c*) physician-diagnosed chronic disease history, including CVD (including revascularization procedures and myocardial infarction), hypertension, and diabetes. The averages of the second and third measures of seated systolic and diastolic BPs on day 1 were used to represent BP levels of a participant. Day 1 fasting glucose was measured by Penn State GCRC central laboratory. CVD was defined by antianginal medication use or a history of CVD. Hypertension was defined by antihypertensive medication use, physician-diagnosed hypertension, systolic BP ≥ 140 mmHg, or diastolic BP ≥ 90 mmHg. Diabetes was defined by antidiabetic medication use, a diagnosis of diabetes by a physician, or fasting glucose > 126 mg/dL. Body mass index (BMI) was defined as the ratio of weight (kilograms) to height squared (meters).

### Statistical analysis

We used distributed lag models (Almon 1965; Pope and Schwartz 1996; Schwartz 2000) under a framework of linear mixed-effects models (Laird and Ware 1982) with a first-order autoregressive covariance structure to assess the autocorrelation corrected regression coefficients between 30-min PM_2.5_ and the HR-corrected QT measures. Residual diagnostics were used to assess the appropriateness of modeling assumptions, and no sizeable departures were detected. In these models, one lag indicated a 30-min separation between the exposure and outcome. Thus, lag 0 indicated the spontaneous relationships between PM_2.5_ and the HR-corrected QT measures, and lag 1 indicated 30 min between the PM_2.5_ and HR-corrected QT measures, and so on. Because QT interval includes QRS duration, with the latter mostly representing the ventricular depolarization process, we analyzed QRS interval in models identical to those for the QT variables. We chose a constrained distributed lag model, the polynomial distributed lag model, to reduce the potential collinearity of PM_2.5_ between individual lags using a second-degree polynomial. Another advantage of the distributed lag model is its ability to provide interpretation of the cumulative effects of the lags included in the model, as well as individual lag effects. Because the PM_2.5_ and ECG variables were assessed in parallel over 48 lags (24 hr), we predetermined to model no more than 10 lags, which allowed us to fit the distributed lag models using at least 80% of the data. We started from the largest number of lags (lag 0–lag 10), and identified significant (*p* < 0.05) cumulative effects of PM_2.5_ on the ECG variables. In this report, the cumulative effect on QTI was significant in the 10-lag model. From this 10-lag significant cumulative-effect model, we reduced the total number of individual lags by back-eliminating the nonsignificant longer lags (e.g., lag 10), one lag at a time until a significant individual lag was identified (lag 7 in this report). We then identified this model as our final model for all ECG outcomes. All results were expressed per 10-μg/m^3^ increase in PM_2.5_. The distributed lag models are summarized in Table 2, where model 1 was adjusted for basic demographic variables and model 2 included an additional adjustment for diabetes, hypertension, and CVD. We repeated model 2 by adjusting for each of the HRV variables to examine the impact of cardiac autonomic modulation on PM_2.5_ and QT associations. These results are summarized in Table 3. In these models, all time-dependent covariables, such as weather and HRV variables, were entered in the model using the same distributed lag structure as the PM_2.5_ variable.

## Results

**Figure 1:**
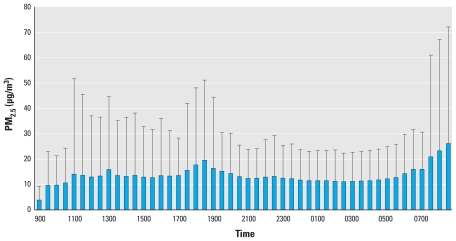
Time-specific PM_2.5_ exposure (mean ± SD) over 24 hr in the APACR study.

**Figure 2:**
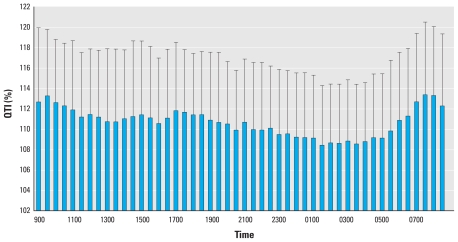
Time-specific QTI (mean ± SD) over 24 hr in the APACR study.

The demographic and CVD risk profiles of the study population are presented in Table 1. The mean age of the participants was 56 years old, 74% were non-Hispanic white, 26% were minorities (including blacks, Hispanics, and Chinese), 59% were female, and 43% had chronic diseases (mostly hypertension). Nine individuals (8.5%) reported being diagnosed with coronary heart disease by a physician > 2 years ago. At the population level, the distributions of both the PM_2.5_ exposure and ventricular repolarization outcome variables are approximately normal. The time of the day-specific distributions of the PM_2.5_ and QTI, as an example of ventricular repolarization measures, are presented in Figures 1 and 2, respectively. Both the PM_2.5_ and QTI showed sufficient variations, both between time points and between individuals, within the 24-hr time frame.

The cumulative effects and individual lag effects of PM_2.5_ on each of the HR-corrected QT measures and QRS duration are summarized in Table 2 as multivariable adjusted regression coefficients (± SE) associated with a 10-μg/m^3^ increment of PM_2.5_ exposure. In summary, the cumulative effect based on lag 0–lag 7 (3.5 hr) on QTI was significant (*p* < 0.01). Examining the individual lag effects, most of the adverse ventricular repolarization effects from direct PM_2.5_ exposure occurred around 3–3.5 hr (lag 6–lag 7) after the elevation of PM_2.5_. The multivariable adjusted regression coefficients for the association between a 10-μg/m^3^ increase in PM_2.5_ and QTI were also statistically significant at lag 0 and lag 1, suggesting both very early- and later-phase responses. Additional adjustment for chronic disease did not change the pattern of association from the models adjusted only for major demographic and weather-related variables. PM_2.5_ was not associated with QRS duration in this population (Table 2).

The final multivariable adjusted PM_2.5_ and QT measures (model 2) in Table 2 were rerun with additional adjustment for HRV variables, using one HRV variable at a time. The HRV-adjusted PM_2.5_ and QT associations are presented in Table 3. In summary, the overall pattern of associations between PM_2.5_ and HR-corrected QT interval measures did not change substantially with additional adjustment for HRV variables as measures of cardiac autonomic modulation. However, the estimated lag 7 PM_2.5_ effects on QTI were attenuated, and the *p*-values from this lag were marginally significant (corresponding *p*-values were > 0.05 but < 0.10). To examine the potential confounding by the circadian variations of both QT variables and the PM_2.5_ exposures, we also stratified our final models (model 2) as daytime versus nighttime for ECG measures (using 2100 hours as a cut off). These stratified models indicated similar patterns of association as those from the 24-hr overall models (data not shown).

We tested the interaction terms between PM_2.5_ and chronic conditions and found no statistical significance at *p* < 0.05 level (data not shown). Therefore, the estimated effects of PM_2.5_ on ventricular repolarization measures did not differ significantly depending on whether a person had previous health problems. We also performed stratified analysis according to chronic disease status, using the models in Table 2, model 1. We found similar associations by chronic disease status (data not shown). It should be noted that the sample size of this study is small, and individuals with chronic conditions consisted mostly of well-controlled hypertensives. The statistical power was limited to detect significant effect modification by chronic disease status.

## Discussion

A large number of epidemiologic studies have found an association between short-term exposure to increased particulate air pollution and CVD morbidity and mortality (Franklin et al. 2007, 2008; Ostro et al. 2006; Zanobetti and Schwartz 2009). However, the mechanisms responsible for such an association have not been fully identified. Previous studies have suggested several promising underlying mechanisms, including cardiac autonomic impairment as measured by lower HRV (Creason et al. 2001; Gold et al. 2000; Liao et al. 1999, 2004; Pope et al. 1999), and ventricular repolarization (Campen et al. 2006; Ghelfi et al. 2008; Henneberger et al. 2005; Lux and Pope 2009; Samet et al. 2009; Yue et al. 2007). Various studies, including patient-based population, panel study, large population-based cohort, controlled exposures, or ambient fixed-location air pollution measures, have indirectly suggested short-term PM effects on cardiac electrophysiology and clinically relevant arrhythmias (Dockery et al. 2005; Elder et al. 2007; Liao et al. 1999, 2004, 2009; Lux and Pope 2009; Park et al. 2005; Yue et al. 2007; Zanobetti et al. 2009; Zhang et al. 2009); these cardiac parameters included HRV, ventricular repolarization, T-wave alternans, myocardium ischemia, and arrhythmias. The actual time course from PM exposure to effects on cardiac repolarization measures has not been investigated systematically in a community-dwelling sample, nor has it been determined whether the PM and ventricular repolarization association would be mediated through its impact on cardiac autonomic modulation. Cavallari et al. reported an early- and a later-phase HRV response, with the early effects at 2 hr and delayed effects at 9–13 hr after exposure (Cavallari et al. 2008).

For the healthy individuals in this community-based study, PM_2.5_ had a significant adverse association with ventricular repolarization, regardless of which HR-corrected QT intervals were used. Specifically, the estimated cumulative effects of PM_2.5_ on QTI were statistically significant, and the direction of point estimates from the other two HR-corrected QTs (QtcB and QTcF) indicated the same anticipated direction as QTI, although the estimated cumulative effects for these latter two QTs were not significant at the *p* < 0.05 level. For the individual lag effects, all three QT measures were consistently associated with PM_2.5_ exposures at lag 6 and lag 7 (approximately 3–3.5 hr after elevated PM_2.5_), with the estimated effect on QTI also showing evidence of an earlier-phase effect (lag 0 and lag 1, approximately within 1 hr of PM exposure elevation). We did not find any association between PM_2.5_ and QRS duration. Therefore, these data support that elevated PM_2.5_ levels can lead to longer ventricular repolarization but have no immediate impact on ventricular depolarization (QRS duration). The apparent effect courses within 3–3.5 hr of elevated PM_2.5_ exposure. The results presented in Table 3 indicate that the PM and QT and PM–QRS association remains unchanged, even after adjusting for HRV variables as potential intermediating factors. These data further suggest that the PM_2.5_ and ventricular repolarization measures were not mediated through adverse effects on cardiac autonomic modulation, at least not effects with the same lag from exposure. Furthermore, the time from exposure to apparent effects was approximately 3–3.5 hr. Considered with other studies that have indicated shorter-term acute PM effects on cardiac electrophysiology and arrhythmias, our current time-course study results are consistent with previous studies and are suggestive of an acute PM_2.5_-mediated disturbance of the ventricular repolarization process, which may contribute to acute cardiac events, particularly arrhythmias and sudden cardiac death. To our knowledge, this is the first study to demonstrate such findings in a community-based sample.

A number of approaches have been developed to estimate ventricular repolarization including the HR-corrected QT intervals that we used in this study—QTI, QTcB, and QTcF. We used these three HR-corrected QT measures because QTcB is the first HR-corrected QT index, and QTcF has similar properties but better HR correction compared with QTcB. Both QTcB and QTcF are widely used in the literature. We also used the less frequently seen QTI because it was reported to be less sensitive to changes in HR and more repeatable than QTcB (Rautaharju et al. 1991). On the other hand, it was suggested that these HR-corrected QT measures should not be used to determine the treatment effects in patients with QT-prolongation syndrome (Rautaharju et al. 2009). However, this study sample represents community-dwelling individuals, and none have long QT syndrome. In effect, we designed this study to investigate the impact of PM on the variation of HR-corrected QT intervals within normal range in relatively healthy individuals. Moreover, these simple HR-corrected QT variations among normal healthy individuals have been associated with significant prediction of future risk of cardiac events (Rautaharju et al. 2006a, 2006b), supportive of our use of these indices.

It should be noted that the effect sizes we estimated in this study are relatively small. For example, for every 10-μg /m^3^ increase in PM_2.5_, the associated cumulative increase in QTI from previous 3.5 hr is only 0.32, corresponding to less than a 1% increase in this variable, which has a mean of 110 and SD of 6.6. For another instance, for every 10-μg /m^3^ increase in lag 7 PM_2.5_, the associated increase in QTcB is only 0.22 msec, corresponding to less than a 1% increase in this variable, which has a mean of 438 msec and SD of 23 msec. Although such small changes in QTI may not be clinically meaningful, it can be argued that the entire population is exposed to PM_2.5_ in the ambient air, and from personal and indoor sources, on a continuous daily basis. Thus, elevated PM_2.5_ levels have a potentially large public health impact. Moreover, the minor effect on QTI estimated in this study was measured in generally healthy individuals. It is possible that PM_2.5_ effects on ventricular repolarization might be greater in individuals with underlying structural heart disease, ischemic heart disease, channelopathies, or drug-induced effects on repolarization. Future studies should target these clinical subgroups likely to be more susceptible to the effects of PM_2.5_, especially those made more vulnerable by residing near sources of PM_2.5_, for example, near highways.

There are several limitations. First, the APACR study excluded smokers and persons with acute cardiac events within the past 6 months. Thus, our findings may not apply to smokers or persons with a recent acute cardiac event. Second, the majority of participants reported that they stayed indoors most of the time during the 24-hr study period, except when they had to travel by automobile. This behavior pattern is reflected in the relatively low levels of exposure to PM_2.5_. In general, our participants had limited indoor exposures, such as secondhand smoking. Thus, we were unable to assess whether exposures at much higher levels would exhibit similar associations. However, we purposely used the personal monitors and real-time Holter system to collect the true individual level exposure and routine ECG data, respectively. We argue that the associations we observed in these individuals are more reflective of their routine exposure and outcome associations. Third, the ECG data from Holter were not collected under a controlled, supine-position setting. Thus, the short-term variation of other factors that may impact the ventricular repolarization cannot be fully accounted for. However, it is not feasible to keep a healthy participant in a supine indoor position for 24 hr. Even if this were achieved, the results from such a study design would likely have limited variation in PM_2.5_ exposure levels, and the data from such a study would not be generalizable to a real-world situation. In contrast, our study captures the range of activities occurring in real life, including time spent outdoors, time spent commuting in an automobile, and various other activities associated with a disease-free, community-dwelling individual. Finally, PM_2.5_ was the only pollutant on which data were collected. The observed associations could be due to other unmeasured pollutants highly correlated with PM_2.5_.

In summary, acute exposure to PM_2.5_ at the individual level is associated with longer HR-corrected QT interval measures, and the time to the apparent effect is about 3–3.5 hr. The estimated effect of PM on ventricular repolarization was independent of major confounding factors and cannot be attributed solely to effects of PM on cardiac autonomic modulation (HRV). There was no association between PM_2.5_ and QRS duration, suggesting no effects on ventricular depolarization. Overall, these findings support that PM may affect ventricular repolarization, and partly through such a mechanism, PM increases cardiovascular risk, such as sudden cardiac death.

## Figures and Tables

**Table 1:** Demographic characteristics and health status of the study population.^a^

		Hypertension, diabetes, or CVD	
Characteristic	All subjects *n* =106	No *n* = 60	Yes *n* = 46
Age (years)	56 ± 7.6	56 ± 8.2	57 ± 6.8
Sex (% male)	41	40	41
Race (% white)	74	72	76
Glucose (mg/dL)	89 ± 25	85 ± 10	94 ± 36
Body mass index (kg/m^2^)	27.71 ± 5.86	26.19 ± 4.31	29.69 ± 6.98
History of coronary heart disease (%)	8.5	0.0	19.6
Hypertension (%)	35	0.0	85
Diabetes (%)	7.6	0.0	17.4
Systolic BP (mmHg)	122 ± 15.7	117 ± 11.9	128 ± 17.93
Diastolic BP (mmHg)	75 ± 9.2	73 ± 8.3	78 ± 9.8
College or higher education (%)	78	73	85
QTI	110.68 ± 6.60	109.98 ± 6.28	111.59 ± 6.90
QTcB (msec)	438.11 ± 23.34	435.33 ± 21.52	441.70 ± 25.05
QTcF (msec)	421.70 ± 21.55	419.44 ± 20.40	424.62 ± 22.62
QRS duration (msec)	93.97 ± 10.78	94.22 ± 10.49	93.65 ± 11.13
PM_2.5_ (μg/m^3^)	13.61 ± 21.59	11.86 ± 14.69	15.87 ± 27.95
Temperature (°C)	22 ± 3.5	22 ± 3.7	22 ± 3.4
Relative humidity (%)	40 ± 12.1	40 ± 12.3	39 ± 11.8

a Results represent means ± SD for continuous variables and percentage (%) for binary variables.

**Table 2:** Regression coefficient (SE) of HR- corrected QT measures associated with 10-μg/m^3^ increment of PM_2.5_ concentration.

	Regression coefficient (SE)	
QT variable/Lags	Model 1^a^	Model 2^b^
QTI		
Lag 0 (same time)	0.08 (0.04)*	0.07 (0.04)*
Lag 1 (30 min)	0.05 (0.02)*	0.04 (0.02)*
Lag 2 (60 min)	0.03 (0.04)	0.02 (0.02)
Lag 3 (90 min)	0.02 (0.03)	0.01 (0.03)
Lag 4 (120 min)	0.02 (0.03)	0.01 (0.03)
Lag 5 (150 min)	0.03 (0.02)	0.02 (0.02)
Lag 6 (180 min)	0.05 (0.03)*	0.05 (0.03)*
Lag 7 (210 min)	0.08 (0.04)*	0.08 (0.04)*
Cumulative	0.37 (0.13)**	0.32 (0.13)**
QTcB (msec)		
Lag 0 (same time)	0.05 (0.08)	0.05 (0.08)
Lag 1 (30 min)	0.02 (0.06)	0.02 (0.06)
Lag 2 (60 min)	0.01 (0.06)	0.003 (0.06)
Lag 3 (90 min)	0.02 (0.07)	0.01 (0.07)
Lag 4 (120 min)	0.04 (0.07)	0.03 (0.07)
Lag 5 (150 min)	0.08 (0.06)	0.08 (0.06)
Lag 6 (180 min)	0.14 (0.06)*	0.14 (0.06)*
Lag 7 (210 min)	0.22 (0.09)**	0.22 (0.08)**
Cumulative	0.59 (0.39)	0.54 (0.39)
QTcF (msec)		
Lag 0 (same time)	−0.05 (0.05)	−0.05 (0.05)
Lag 1 (30 min)	−0.04 (0.04)	−0.04 (0.04)
Lag 2 (60 min)	−0.02 (0.04)	−0.02 (0.04)
Lag 3 (90 min)	−0.002 (0.04)	−0.004 (0.04)
Lag 4 (120 min)	0.02 (0.04)	0.02 (0.04)
Lag 5 (150 min)	0.04 (0.04)	0.04 (0.04)
Lag 6 (180 min)	0.06 (0.04)	0.06 (0.04)
Lag 7 (210 min)	0.09 (0.05)*	0.09 (0.05)*
Cumulative	0.11 (0.25)	0.09 (0.25)
QRS duration (msec)		
Lag 0 (same time)	−0.002 (0.014)	−0.002 (0.014)
Lag 1 (30 min)	0.001 (0.009)	0.001 (0.009)
Lag 2 (60 min)	0.002 (0.012)	0.002 (0.012)
Lag 3 (90 min)	−0.001 (0.015)	−0.001 (0.015)
Lag 4 (120 min)	−0.005 (0.016)	−0.005 (0.016)
Lag 5 (150 min)	−0.012 (0.017)	−0.012 (0.017)
Lag 6 (180 min)	−0.021 (0.019)	−0.021 (0.019)
Lag 7 (210 min)	−0.033 (0.026)	−0.032 (0.026)
Cumulative	−0.070 (0.092)	−0.068 (0.092)

a Model 1 adjusted for age, sex, race, temperature, and relative humidity.

b Model 2 adjusted for model 1 covariables, plus diabetes, hypertension, and CVD.

* *p* < 0.05.

** *p* < 0.01.

**Table 3:** Regression coefficient (SE) of HR-corrected QT measures associated with 10-μg/m^3^ increment of PM_2.5_ with additional adjustment for HRV variables.^a^

	QTI				QTcB (msec)				QTcF (msec)			
	HF	LF	SDNN	RMSSD	HF	LF	SDNN	RMSSD	HF	LF	SDNN	RMSSD
Lag 0 (same time)	0.08 (0.04)**	0.07 (0.04)**	0.09 (0.04)**	0.08 (0.04)**	0.05 (0.08)	0.05 (0.08)	0.07 (0.08)	0.05 (0.08)	−0.05 (0.05)	−0.05 (0.05)	−0.05 (0.05)	−0.05 (0.05)
Lag 1 (30 min)	0.04 (0.02)*	0.04 (0.02)*	0.04 (0.02)*	0.04 (0.02)*	0.01 (0.06)	0.01 (0.06)	0.02 (0.06)	−0.01 (0.06)	−0.04 (0.04)	−0.04 (0.04)	−0.04 (0.04)	−0.04 (0.04)
Lag 2 (60 min)	0.01 (0.02)	0.01 (0.02)	0.01 (0.02)	0.02 (0.02)	−0.01 (0.06)	−0.01 (0.06)	−0.01 (0.06)	−0.03 (0.06)	−0.02 (0.04)	−0.02 (0.04)	−0.02 (0.04)	−0.03 (0.04)
Lag 3 (90 min)	−0.001 (0.03)	0.001 (0.03)	−0.01 (0.03)	0.01 (0.03)	−0.003 (0.07)	0.0004 (0.07)	−0.01 (0.07)	−0.03 (0.06)	0.003 (0.04)	0.004 (0.04)	−0.002 (0.04)	−0.01 (0.04)
Lag 4 (120 min)	−0.001 (0.03)	0.001 (0.03)	−0.01 (0.03)	0.01 (0.03)	0.02 (0.07)	0.03 (0.07)	0.02 (0.07)	−0.004 (0.07)	0.03 (0.04)	0.03 (0.04)	0.02 (0.04)	0.02 (0.04)
Lag 5 (150 min)	0.01 (0.03)	0.01 (0.03)	0.004 (0.03)	0.02 (0.03)	0.08 (0.07)	0.08 (0.07)	0.07 (0.07)	0.05 (0.07)	0.05 (0.04)	0.06 (0.04)	0.05 (0.04)	0.04 (0.04)
Lag 6 (180 min)	0.03 (0.03)	0.04 (0.03)	0.03 (0.03)	0.04 (0.03)	0.15 (0.07)**	0.15 (0.07)**	0.15 (0.07) **	0.13 (0.07)**	0.08 (0.04)**	0.08 (0.04)**	0.08 (0.04)**	0.08 (0.04)*
Lag 7 (210 min)	0.07 (0.04)*	0.07 (0.04)*	0.07 (0.04)*	0.08 (0.04)*	0.25 (0.09)#	0.25 (0.09)#	0.25 (0.09)#	0.24 (0.09)#	0.12 (0.05)**	0.12 (0.05)**	0.12 (0.05)**	0.11 (0.05)**
Cumulative	0.24 (0.14)*	0.25 (0.14)*	0.23 (0.14)*	0.29 (0.13)**	0.56 (0.40)	0.57 (0.40)	0.56 (0.40)	0.39 (0.40)	0.17 (0.25)	0.18 (0.25)	0.17 (0.25)	0.11 (0.25)

a Adjusted for age, sex, race, temperature, relative humidity, diabetes, hypertension, CVD, and each of the HRV indices.

* *p* < 0.1.

** *p* < 0.05.

# *p* < 0.01.
